# An electromechanical stimulation regulating model with flexoelectric effect of piezoelectric laminated micro-beam for cell bionic culture

**DOI:** 10.1038/s41598-024-56708-9

**Published:** 2024-03-13

**Authors:** Wei-Feng Rao, Ya-Wen Wang, An-Qing Li, Sha-Sha Zhou, Zu-Mei Zheng

**Affiliations:** 1 School of Mechanical Engineering, Qilu University of Technology (Shandong Academy of Sciences), Jinan, 250353 People’s Republic of China; 2 Shandong Institute of Mechanical Design and Research, Jinan, 250031 People’s Republic of China

**Keywords:** Piezoelectric effect, Flexoelectric effect, Electromechanical stimulation, Laminated micro-beam, Cell bionic culture, Engineering, Mechanical engineering

## Abstract

Cell bionic culture requires the construction of cell growth microenvironments. In this paper, mechanical force and electrical stimulations are applied to the cells cultured on the surface of the piezoelectric laminated micro-beam driven by an excitation voltage. Based on the extended dielectric theory, the electromechanical microenvironment regulating model of the current piezoelectric laminated micro-beam is established. The variational principle is used to obtain the governing equations and boundary conditions. The differential quadrature method and the iterative method are used to solve two boundary value problems for cantilever beams and simply supported beams. In two cases, the mechanical force and electrical stimulations applied to the cells are analyzed in detail and the microscale effect is investigated. This study is meaningful for improving the quality of cell culture and promoting the cross-integration of mechanics and biomedicine.

## Introduction

In the medical field, drug research and development need to go through a long process of animal and clinical experiments. And the discomfort of drugs screened by animal models in humans is as high as 95%. It is difficult to accurately predict the effect of drugs on the human body, either in animal experiments or in vitro experiments. It is imperative to create a new drug screening tool that is more precise, closer to human physiological functions, and can take the place of animal experiments. The organ-on-a-chip was developed with such a goal. It is a multi-channel cell culture device composed of two parts. One is the ontology established by the corresponding cells according to the proportion and order in the solid organ. The second is the microenvironment in the tissue, which mainly refers to other cells in the tissue, secretory substances, external forces, etc.^1,2^. The construction of organ physiological microsystems on organs-on-chips can simulate the physiological and pathological activities of human organs in vitro^3^. With this technology, researchers can not only use it to witness and study various biological behaviors of organisms but also predict the response of the human body to drug stimulation^4,5^. Therefore, it is widely used in disease research, research and development of new drugs^6,7^.

Many cells in the human body are in a dynamic, physically stimulating microenvironment. The bionic culture of cells in the organ-on-a-chip, which represents the physiological state of the body, can significantly enhance the in vivo organ simulation quality of the organ-on-a-chip. Kizilkurtlu et al.^8^ simulated the respiratory movement mechanism of the human lung by pulling and pressing PDMS membrane under vacuum, and constructed the traction stress microenvironment of the human lung. Langerak et al.^9^ designed a new intestinal chip, which inoculated intestinal cells under a single shear stress and optimized cell differentiation by regulating shear stress to improve the function of monolayer cells. Ribeiro^10^ analyzed the effect of piezoelectric polyvinylidene fluoride on bone formation, and its osteogenic properties were tested by new bone formation in rats. Many studies have shown that the electrical stimulation provided to cells by the inverse piezoelectric effect of piezoelectric polyvinylidene fluoride enhances bone regeneration in vivo. Tang^11^ cultured cells on the nanocomposite film, and the magnetic powder was doped on the film. The potential generated by the external magnetic field induced the growth of the cells, and the interaction between the cells and the material during the specific cell growth period was used to effectively achieve the required osteogenic differentiation. Hoop^12^ pointed out that electrical stimulation plays an important role in the regeneration of various functions of soft tissue, and studies have found that the charge generated by polyvinylidene fluoride in the sound field can form an electrical environment conducive to the differentiation of nerve cells. The above studies have shown that the construction of an electromechanical stimulation microenvironment for cell growth is an important issue in the bionic culture of cells on organs-on-chips. However, the current research only focuses on a single physical stimulation, and there is a lack of related research that considers both mechanical and electrical physical stimulation. This paper attempts to use the periodic deformation of the piezoelectric polymer film to apply mechanical force stimulation to the cells, and at the same time use the electric potential induced by the inverse piezoelectric effect of the piezoelectric polymer film to give the cells electrical stimulation so as to achieve the electromechanical physiology microenvironment required for cell bionic culture.

Applying accurate mechanical force and electrical stimulation to cells requires mastering the dynamic characteristics of piezoelectric polymer films. Based on the Bernoulli–Euler beam, Przybylski^13^ established a bending vibration model under external load and piezoelectric drive. Karimipour^14^ studied the influence of material length scale parameters on the vibration response of various geometric shapes. Kapuria^15^ established a circular plate model with an isotropic elastic core in the middle and a piezoelectric layer on the surface. The nonlinear static and dynamic responses of laminated thin circular plates under axisymmetric electrical loads were analyzed. Karimipour^16^ studied the effects of size effect, electrical load (DC and AC voltage) and excitation frequency on the static and dynamic response, critical AC voltage and dynamic stability of the microplate. Seo^17^ designed a tactile stimulation resonant actuator based on a piezoelectric polymer film. An air chamber is placed on the PVDF film for indirect piezoelectric drive, which can achieve large displacement at low input voltage. The resonant driver can obtain a fast response when stimulating human skin and can be used for tactile output devices.

Moreover, many researchers demonstrate that the electromechanical coupling performance of components at the micro-nanoscale shows size dependence^18^. Many studies have shown that this phenomenon is caused by the flexoelectric effect. The flexoelectric effect is a new higher-order electromechanical coupling effect discovered in recent two decades. The positive flexoelectric effect induces polarization by strain gradient, and the inverse flexoelectric effect generates mechanical deformation stress for the polarization gradient^19^. Tadi Beni et al.^20^ established models of double-clamped nano-bridge and clamped-free nano-cantilever, and found that the physical response of nanostructures is size-dependent. Li et al.^21^ established a size-dependent three-layer micro-beam model including flexoelectric dielectric layer and studied the static bending and free vibration problems of cantilever beams and simply supported beams. Karimipour and Tadi Beni^22^ used the nonlocal theory to establish a laminated composite toroid shell model to study the nonlinear forced vibration of composite shallow nano toroidal shell segment. Based on the general formula of flexoelectric theory, Li et al.^23^ established a model of flexoelectric circular micro-plates and solved the static bending problems of simply supported axisymmetric circular micro-plates. Nguyen^24^ established a micro-beam model considering the dynamic flexoelectric effect and studied the free vibration of a simply supported beam. Thai et al.^25^ established a piezoelectric-flexoelectric bending beam model based on couple stress and investigated the nonlinear vibration behaviors. Karimipour^26^ considered size effects in the linear three-dimensional elasticity analysis of micro-tori and established an isotropic annular shell model. The influence of length scale parameter (related to material microstructures) on the mechanical responses was studied. Yan and Jiang^27^ studied the flexoelectric effect in static bending and free vibration of piezoelectric nanobeams. The flexoelectric effect has an important influence on the electromechanical coupling performance of piezoelectric components at the micro-nanoscale, and it is an important factor that must be considered in the study of the dynamic characteristics of piezoelectric micro-components^28,29^.

This paper mainly studies the bending characteristics of three-layer piezoelectric micro-beams, which are used to construct the electromechanical stimulation microenvironment of cell bionic culture. Both the piezoelectric effect and the flexoelectric effect are considered in this study. This article is organized as follows. Based on the extended dielectric theory, the second section mainly describes the establishment of the size-dependent theoretical model of the laminated micro-beam. According to the boundary conditions of the driving layer and the response layer of laminated micro-beam, the corresponding polarization and electric potential are solved respectively. The mechanical governing equations and boundary conditions are derived by the variational principle and discretized according to the differential quadrature method. In the third section, the numerical values of the cantilever beam and the simply supported beam are solved, the mechanical force and electrical stimulations applied to the cells are analyzed in detail and the microscale effect is investigated. Finally, conclusion is summarized in the fourth section.

## The electromechanical stimulation regulating model of piezoelectric laminated micro-beam

**Figure 1:**
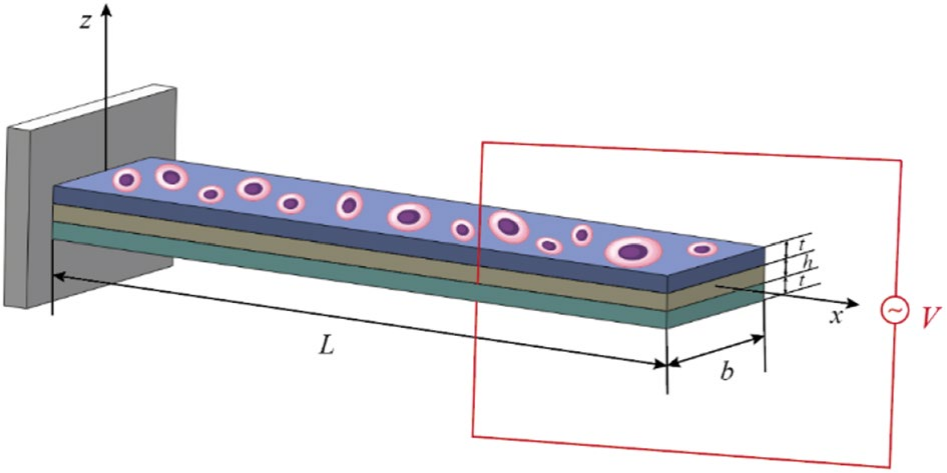
Schematic diagram of three-layer micro-beams for cell bionic culture.

In order to construct the electromechanical stimulation microenvironment of cell bionic culture, a laminated micro-beam structure is designed in this paper, as shown in Fig. 1, in which the laminated micro-beam includes three layers: a response layer, an elastic layer, and a driving layer from top to bottom. Polyvinylidene fluoride trifluoroethylene polymer (P(VDF-TrFE)) with excellent biocompatibility can be selected as the response layer and the driving layer, and polydimethylsiloxane (PDMS) is selected as the intermediate elastic layer. The excitation voltage *V* is applied to the driving layer. Under the action of the piezoelectric effect, the driving layer drives the overall bending deformation of the laminated micro-beam and stimulates the cells cultured on the surface of the response layer with mechanical force. Meanwhile, the response layer induces electric potential through the inverse piezoelectric effect and stimulates the cells cultured on the surface of the response layer with electrical stimulation. In this way, the electromechanical stimulation microenvironment of cell bionic culture is constructed on the surface of the response layer.

In order to set up the theoretical model of the laminated micro-beam structure, the Cartesian coordinate system shown in Fig. 1 is used. The *x–y* plane coincides with the middle layer of the laminated micro-beam, and the *x* direction is along the axial direction of the beam. It is assumed that the length of the laminated micro-beam is *L*, the width is *b*, both the thickness of the driving layer and the response layer is *t*, and the thickness of the elastic layer is *h*. Using the basic assumption of the Bernoulli–Euler beam, the overall displacement field of the micro-beam can be expressed as

1 $$ u = - z\frac{{{\text{ d}}w}}{{{\text{ d}}x}},v = 0,w = w(x) $$

in which *u*, *v,* and *w* are the displacement components of the beam along the *x*, *y,* and *z* directions, respectively. In addition, it is assumed that the polarization is only induced along the thickness direction of the beam.

2 $$ P_{x} = 0,\quad P_{y} = 0,\quad P_{z} = P_{z} (x,z) $$

where *P*_*x*_, *P*_*y*_, and *P*_*z*_ are the polarization components of the micro-beam along the *x*, *y,* and *z* directions, respectively.

In order to describe the size dependence of the electromechanical coupling performance of microscale components, the extended dielectric theory, considering the influence of strain gradient and polarization gradient, is applied in this paper. Compared with the piezoelectric theory, in addition to the traditional elastic term and piezoelectric term, this theory also introduces the coupling term of strain gradient and polarization reflecting the positive flexoelectric effect, the coupling term of polarization gradient and strain reflecting the inverse flexoelectric effect, the coupling term of strain gradient and strain gradient and the coupling term of polarization gradient and polarization gradient reflecting the higher-order effect. The internal energy density of the extended dielectric theory can be expressed as^30,31^

3 $$ U = \frac{1}{2}c_{ijkl} \varepsilon_{ij} \varepsilon_{kl} + \frac{1}{2}g_{ijklmn} \eta_{ijk} \eta_{lmn} + \frac{1}{2}a_{ij} P_{i} P_{j} + \frac{1}{2}b_{ijkl} Q_{ij} Q_{kl} + d_{ijk} P_{i} \varepsilon_{jk} + f_{ijkl} (P_{i} \eta_{jkl} - Q_{ij} \varepsilon_{kl} ) $$

where *ε*_*ij*_ and *P*_*i*_ are strain tensor and polarization vector, respectively; *η*_*ijk*_ and *Q*_*ij*_ are strain gradient tensor and polarization gradient tensor, respectively; and *c*_*ijkl*_, *a*_*ij*_, and *d*_*ijk*_ are elastic constant, dielectric coefficient and piezoelectric coefficient tensors, respectively. The material property tensors *g*_*ijklmn*_ and *b*_*ijkl*_ represent the higher-order elastic effect and electric field effect, respectively, and *f*_*ijkl*_ represents the flexoelectric coupling tensor. The present theory considers the piezoelectric effect, the flexoelectric effect, the stiffening effect of strain gradient and the high-order electric field effect of polarization gradient can reduce to other simplified theory by deleting certain effects. For example, when the terms associated with strain gradient and polarization gradient are ignored, the present theory will reduce to the traditional piezoelectric theory.

The strain tensor, the strain gradient tensor, and the polarization gradient tensor are defined, respectively, as

4 $$ \varepsilon_{ij} = \frac{1}{2}(u_{i,j} + u_{j.i} ),\quad \eta_{ijk} = \varepsilon_{jk,i} ,\quad Q_{ij} = P_{i,j} $$

with *u*_*i*_ denoting the displacement vector, and the comma represents the differential relative to the coordinate. The constitutive equation can be expressed as^32^

5 $$ \sigma_{ij} = c_{ijkl} \varepsilon_{kl} + d_{kij} P_{k} - f_{klij} Q_{kl} $$

6 $$ \tau_{ijk} = g_{ijklmn} \eta_{lmn} + f_{lijk} P_{l} $$

7 $$ E_{i} = a_{ij} P_{j} + d_{ijk} \varepsilon_{jk} + f_{ijkl} \eta_{jkl} $$

8 $$ V_{ij} = b_{ijkl} Q_{kl} - f_{ijkl} \varepsilon_{kl} $$

in which *σ*_*ij*_ is the stress tensor, *τ*_*ijk*_ is the higher-order stress, *E*_*i*_ is the electric field, and *V*_*ij*_ is the higher-order electric field.

Substituting the displacement components of Eq. (1) into Eq. (4), and considering the geometric nonlinearity of the large deformation of the micro-beam, the non-zero term of the strain component can be obtained as

9 $$ \varepsilon_{xx} = - z\frac{{d^{2} w}}{{dx^{2} }} + \frac{1}{2}\left( {\frac{dw}{{dx}}} \right)^{2} $$

Since the thickness of the beam is much smaller than its length, the strain gradient in the axial direction is negligible compared to the strain gradient along the thickness direction. Therefore, this paper only considers the flexoelectric effect induced by the strain gradient in the thickness direction. Combining Eq. (9) and Eq. (4), the non-zero term of the strain gradient tensor along the thickness direction is

10 $$ \eta_{zxx} = - \frac{{{\text{ d}}^{2} w}}{{{\text{ d}}x^{2} }} $$

Substituting strain and strain gradient into the constitutive Eqs. (5)–(8), the stress tensor, the higher-order stress, the electric field, and the higher-order electric field are obtained as follows

11 $$ \sigma_{xx} = c_{1111} \left[ { - z\frac{{{\text{ d}}^{2} w}}{{{\text{ d}}x^{2} }} + \frac{1}{2}\left( {\frac{{{\text{ d}}w}}{{{\text{ d}}x}}} \right)^{2} } \right] + d_{311} P_{z} - f_{3311} P_{z,z} $$

12 $$ \tau_{zxx} = - g_{311311} \frac{{{\text{ d}}^{2} w}}{{{\text{ d}}x^{2} }} + f_{3311} P_{z} $$

13 $$ E_{{_{z} }} = a_{33} P_{z} + d_{311} \left[ { - z\frac{{{\text{ d}}^{2} w}}{{{\text{ d}}x^{2} }} + \frac{1}{2}\left( {\frac{{{\text{ d}}w}}{{{\text{ d}}x}}} \right)^{2} } \right] - f_{3311} \frac{{{\text{ d}}^{2} w}}{{{\text{ d}}x^{2} }} $$

14 $$ V_{zz} = b_{3333} P_{z,z} - f_{3311} \left[ { - z\frac{{{\text{ d}}^{2} w}}{{{\text{ d}}x^{2} }} + \frac{1}{2}\left( {\frac{{{\text{ d}}w}}{{{\text{ d}}x}}} \right)^{2} } \right] $$

According to the general governing equation of the extended dielectric theory^30^, the governing equation of the electric field of beam bending is

15 $$ a_{33} P_{z} + d_{311} \left[ { - z\frac{{{\text{ d}}^{2} w}}{{{\text{ d}}x^{2} }} + \frac{1}{2}\left( {\frac{{{\text{ d}}w}}{{{\text{ d}}x}}} \right)^{2} } \right] - 2f_{3311} \frac{{{\text{ d}}^{2} w}}{{{\text{ d}}x^{2} }} - b_{3333} P_{z,zz} + \varphi_{,z} = 0 $$

16 $$ - \varepsilon_{0} \varphi_{,zz} + P_{z,z} { = }0 $$

where *ε*_*0*_ is the dielectric constant of vacuum and *φ* is the potential of Maxwell's self-field.

For the driving layer, its boundary conditions satisfy

17 $$ V_{zz} |_{{z = - \left( {\frac{h}{2} + t} \right)\quad }} = 0,\quad V_{zz} |_{{z = - \frac{h}{2}\quad }} = 0,\quad \varphi |_{{z = - \left( {\frac{h}{2} + t} \right)}} = V,\quad \varphi |_{{z = - \frac{h}{2}}} = 0\quad  $$

The boundary conditions for the response layer are

18 $$ V_{zz} |_{{z = \frac{h}{2}\quad }} = 0,\quad V_{zz} |_{{z = \frac{h}{2}{ + }t}} = 0,\quad D_{z} |_{{z = \frac{h}{2}\quad }} = 0,\quad D_{z} |_{{z = \frac{h}{2}{ + }t}} = 0 $$

Combining the governing Eqs. (15), (16) and the boundary conditions (17), the polarization *P*_*z*_^*d*^ and the electric potential *φ*_*d*_ of the driving layer can be obtained as

19 $$ \begin{aligned} P_{z}^{d} & = \left( { - \frac{{f_{3311} }}{{2b_{3333} \lambda }}\frac{{e^{\lambda (h/2 + z)} (1 - e^{\lambda t} ) + e^{ - \lambda (h/2 + z)} (1 - e^{ - \lambda t} )}}{{e^{ - \lambda t} - e^{\lambda t} }} - \frac{{d_{311} }}{{2a_{33} }}} \right)\left( {\frac{{{\text{ d}}w}}{{{\text{ d}}x}}} \right)^{2} \\ & \quad - \frac{1}{{b_{3333} \lambda^{2} }}\left[ {\left( {\frac{{d_{311} }}{\lambda } - \frac{{f_{3311} \lambda h}}{2}} \right)\frac{{e^{\lambda (h/2 + z)} (1 - e^{\lambda t} ) + e^{ - \lambda (h/2 + z)} (1 - e^{ - \lambda t} )}}{{e^{ - \lambda t} - e^{\lambda t} }}} \right. \\ & \quad + f_{3311} \lambda t\frac{{e^{\lambda (h/2 + z)} + e^{ - \lambda (h/2 + z)} }}{{e^{ - \lambda t} - e^{\lambda t} }} - d_{311} z \\ & \quad \left. { - \frac{{2f_{3311} (1 + 2b_{3333} \varepsilon_{0} \lambda^{2} ) - d_{311} (h + t)}}{{2a_{33} \varepsilon_{0} }}} \right]\frac{{{\text{ d}}^{2} w}}{{{\text{ d}}x^{2} }} + \frac{V}{{a_{33} t}} \\ \end{aligned} $$

20 $$ \begin{aligned} \varphi_{d} & = - \frac{{f_{3311} }}{{2b_{3333} \varepsilon_{0} \lambda^{2} }}\left( {\frac{{e^{\lambda (h/2 + z)} (1 - e^{\lambda t} ) - e^{ - \lambda (h/2 + z)} (1 - e^{ - \lambda t} )}}{{e^{ - \lambda t} - e^{\lambda t} }} - 1} \right)\left( {\frac{{{\text{ d}}w}}{{{\text{ d}}x}}} \right)^{2} \\ & \quad - \frac{1}{{b_{3333} \varepsilon_{0} \lambda^{2} }}\left[ {\left( {\frac{{d_{311} }}{{\lambda^{2} }} - \frac{{f_{3311} h}}{2}} \right)\frac{{e^{\lambda (h/2 + z)} (1 - e^{\lambda t} ) - e^{ - \lambda (h/2 + z)} (1 - e^{ - \lambda t} )}}{{e^{ - \lambda t} - e^{\lambda t} }}} \right. \\ & \quad - f_{3311} t\frac{{e^{ - \lambda (h/2 + z)} - e^{\lambda (h/2 + z)} }}{{e^{ - \lambda t} - e^{\lambda t} }} + \left( {f_{3311} - \frac{{d_{311} {(}h + t)}}{2}} \right)z - \frac{{d_{311} }}{2}\left( {\frac{{h^{2} }}{4} + \frac{ht}{2}} \right) \\  & \quad \left. -{ \frac{{d_{311} }}{{\lambda^{2} }} - \frac{{d_{311} z^{2} }}{2} + f_{3311} h} \right]\frac{{{\text{ d}}^{2} w}}{{{\text{ d}}x^{2} }} - \frac{2z + h}{{2t}}V \\ \end{aligned} $$

where the parameter *λ* is defined as

21 $$ \lambda = \sqrt {\frac{{\varepsilon_{0} a_{33} + 1}}{{\varepsilon_{0} b_{33} }}} $$

Combining the governing Eqs. (15), (16) and the boundary conditions (18), the polarization *P*_*z*_^*s*^ and the electric potential *φ*_*s*_ of the response layer can be solved as

22 $$ \begin{aligned} P_{z}^{s} & = \frac{1}{{b_{3333} \lambda^{2} }}\left[ {f_{3311} \lambda t\frac{{e^{ - \lambda (h/2 - z)} + e^{\lambda (h/2 - z)} }}{{e^{ - \lambda t} - e^{\lambda t} }} + d_{311} z + 2f_{3311} } \right. \\ & \quad \left. { - \left( {\frac{{d_{311} }}{\lambda } + \frac{{f_{3311} \lambda h}}{2}} \right)\frac{{e^{ - \lambda (h/2 - z + t)} - e^{\lambda (h/2 - z + t)} + e^{\lambda (h/2 - z)} - e^{ - \lambda (h/2 - z)} }}{{e^{ - \lambda t} - e^{\lambda t} }}} \right]\frac{{{\text{ d}}^{2} w}}{{{\text{ d}}x^{2} }} \\ & \quad \left. { + \frac{1}{{2b_{3333} \lambda^{2} }}[f_{3311} \lambda \frac{{e^{ - \lambda (h/2 - z + t)} + e^{\lambda (h/2 - z + t)} - e^{\lambda (h/2 - z)} - e^{ - \lambda (h/2 - z)} }}{{e^{ - \lambda t} - e^{\lambda t} }} - d_{311} } \right]\left( {\frac{{{\text{ d}}w}}{{{\text{ d}}x}}} \right)^{2} \\ \end{aligned} $$

23 $$ \begin{aligned} \varphi_{s} & = \frac{1}{{b_{3333} \varepsilon_{0} \lambda^{2} }}\left[ {f_{3311} t\frac{{e^{ - \lambda (h/2 - z)} - e^{\lambda (h/2 - z)} }}{{e^{ - \lambda t} - e^{\lambda t} }} + \frac{{d_{311} }}{2}\left( {\frac{2}{{\lambda^{2} }} - (\frac{h}{2} + t)^{2} + z^{2} } \right) - f_{3311} \left( {\frac{h}{2} + t - 2z} \right)} \right. \\ & \quad \left. { + \left( {\frac{{d_{311} }}{{\lambda^{2} }} + \frac{{f_{3311} h}}{2}} \right)\frac{{e^{\lambda (h/2 - z + t)} - e^{ - \lambda (h/2 - z + t)} + e^{ - \lambda (h/2 - z)} - e^{\lambda (h/2 - z)} }}{{e^{ - \lambda t} - e^{\lambda t} }}} \right]\frac{{{\text{ d}}^{2} w}}{{{\text{ d}}x^{2} }} \\ & \quad + \frac{1}{{2b_{3333} \varepsilon_{0} \lambda^{2} }}\left[ {f_{3311} \frac{{e^{ - \lambda (h/2 - z + t)} - e^{\lambda (h/2 - z + t)} + e^{\lambda (h/2 - z)} - e^{ - \lambda (h/2 - z)} }}{{e^{ - \lambda t} - e^{\lambda t} }}} \right. \\ & \quad \left. { + d_{311} \left( {\frac{h}{2} + t - z} \right) - f_{3311} } \right]\left( {\frac{{{\text{ d}}w}}{{{\text{ d}}x}}} \right)^{2} \\ \end{aligned} $$

The function of the electric enthalpy density with extended dielectric theory can be written as

24 $$ H = U - \frac{1}{2}\varepsilon_{0} \varphi_{,z} \varphi_{,z} + \varphi_{,z} P_{z} $$

The work, *W*, of the transverse external load *q* is defined as

25 $$ W = b\int_{0}^{L} {qwdx} $$

According to the principle of electric enthalpy variation

26 $$ \delta \left( {\int_{\Omega }^{{}} {Hd\Omega } - W} \right) = 0 $$

The mechanical governing equations of laminated piezoelectric micro-beams can be obtained from Eqs. (24)–(26).

27 $$ a_{1} \frac{{{\text{ d}}^{4} w}}{{{\text{ d}}x^{4} }} + a_{2} \left( {\frac{{{\text{ d}}w}}{{{\text{ d}}x}}} \right)^{2} \frac{{{\text{ d}}^{2} w}}{{{\text{ d}}x^{2} }} - \frac{{Vd_{311} }}{{a_{33} }}\frac{{{\text{ d}}^{2} w}}{{{\text{ d}}x^{2} }} + a_{3} \left( {\frac{{{\text{ d}}^{2} w}}{{{\text{ d}}x^{2} }}} \right)^{2} = q $$

The mechanical boundary conditions are

28 $$ \left[ { - a_{1} \frac{{{\text{ d}}^{3} w}}{{{\text{ d}}x^{3} }} - a_{4} \left( {\frac{{{\text{ d}}w}}{{{\text{ d}}x}}} \right)^{3} + \frac{{Vd_{311} }}{{a_{33} }}\frac{{{\text{ d}}w}}{{{\text{ d}}x}} - a_{3} \frac{{{\text{ d}}w}}{{{\text{ d}}x}}\frac{{{\text{ d}}^{2} w}}{{{\text{ d}}x^{2} }}} \right]\delta w|_{0}^{L} = 0 $$

29 $$ \left[ {a_{5} \frac{{{\text{ d}}^{2} w}}{{{\text{ d}}x^{2} }} + a_{6} \left( {\frac{{{\text{ d}}w}}{{{\text{ d}}x}}} \right)^{2} + \frac{{Vd_{311} }}{{2a_{33} }}(h + t) - \frac{{Vf_{3311} }}{{a_{33} }}} \right]\delta \frac{{{\text{ d}}w}}{{{\text{ d}}x}}|_{0}^{L} = 0 $$

where *a*_*1*_ ~ *a*_*6*_ are shown in the Appendix A.

The present model includes piezoelectric effect, flexoelectric effect, the mechanical effect of strain gradient and the electrical effect of polarization gradient. When the piezoelectric effect is neglected by letting *d*_311_ = 0, the present model will reduce to that of flexoelectric theory. When the flexoelectric effect is neglected by letting *f*_3311_ = 0, the present model will reduce to that of piezoelectric strain gradient theory.

Furthermore, the present nonlinear bending beam model can be expressed in dimensionless form when dimensionless parameters are introduced.

30 $$ \overline{w} = \frac{w}{h},\quad \xi = \frac{x}{L},\quad \overline{q} = \frac{{qL^{4} }}{{a_{1} h}},\quad \overline{V}_{p} = \frac{{d_{311} L^{2} }}{{a_{1} a_{33} }}V,\quad \overline{V}_{f} = \frac{{f_{3311} L^{2} }}{{a_{5} a_{33} h}}V $$

The dimensionless governing equation is expressed as

31 $$ \frac{{{\text{ d}}^{4} \overline{w} }}{{{\text{ d}}\xi^{4} }} + K_{1} \left( {\frac{{{\text{ d}}\overline{w} }}{{{\text{ d}}\xi }}} \right)^{2} \frac{{{\text{ d}}^{2} \overline{w} }}{{{\text{ d}}\xi^{2} }} - \overline{V}_{p} \frac{{{\text{ d}}^{2} \overline{w} }}{{{\text{ d}}\xi^{2} }} + K_{2} \left( {\frac{{{\text{ d}}^{2} \overline{w} }}{{{\text{ d}}\xi^{2} }}} \right)^{2} = \overline{q} $$

The dimensionless boundary conditions are

32 $$ \left[ { - \frac{{{\text{ d}}^{3} \overline{w} }}{{{\text{ d}}\xi^{3} }} - K_{3} \left( {\frac{{{\text{ d}}\overline{w} }}{{{\text{ d}}\xi }}} \right)^{3} + \overline{V}_{p} \frac{{{\text{ d}}\overline{w} }}{{{\text{ d}}\xi }} - K_{2} \frac{{{\text{ d}}\overline{w} }}{{{\text{ d}}\xi }}\frac{{{\text{ d}}^{2} \overline{w} }}{{{\text{ d}}\xi^{2} }}} \right]\delta \overline{w} |_{0}^{1} = 0 $$

33 $$ \left[ {\frac{{{\text{ d}}^{2} \overline{w} }}{{{\text{ d}}\xi^{2} }} + K_{4} \left( {\frac{{{\text{ d}}\overline{w} }}{{{\text{ d}}\xi }}} \right)^{2} + K_{5} \overline{V}_{p} - \overline{V}_{f} } \right]\delta \frac{{{\text{ d}}\overline{w} }}{{{\text{ d}}\xi }}|_{0}^{1} = 0 $$

where

34 $$ K_{1} = \frac{{a_{2} h^{2} }}{{a_{1} }},\quad K_{2} = \frac{{a_{3} h}}{{a_{1} }},\quad K_{3} = \frac{{a_{4} h^{2} }}{{a_{1} }},\quad K_{4} = \frac{{a_{6} h}}{{a_{5} }},\quad K_{5} = \frac{{a_{1} (h + t)}}{{2a_{5} h}} $$

The differential quadrature method^33^ is used to solve the nonlinear bending equations, where the *k*-order partial derivative of the function *f*(*x*) with respect to *x* at any sampling point can be approximated by the weighted linear sum of the function values at all discrete points.

35 $$ \frac{{{\text{ d}}^{k} f(x_{i} )}}{{{\text{ d}}x^{k} }} = \sum\limits_{j = 1}^{N} {A_{ij}^{(k)} f(x_{j} )} $$

where *N* is the total number of discrete points, and $A_{ij}^{(k)}$ is the *k*-order weighted coefficient matrix.

By combining Eq. (35) with (31)–(33), the governing equations is discretized as

36 $$ \begin{aligned} & \sum\limits_{j = 1}^{N} {A_{ij}^{(4)} \overline{w}_{j} } + K_{1} \sum\limits_{j = 1}^{N} {A_{ij}^{(1)} \overline{w}_{j} } \sum\limits_{j = 1}^{N} {A_{ij}^{(1)} \overline{w}_{j} } \sum\limits_{j = 1}^{N} {A_{ij}^{(2)} \overline{w}_{j} } \\ & \quad - \overline{V}_{p} \sum\limits_{j = 1}^{N} {A_{ij}^{(2)} \overline{w}_{j} } + K_{2} \sum\limits_{j = 1}^{N} {A_{ij}^{(2)} \overline{w}_{j} } \sum\limits_{j = 1}^{N} {A_{ij}^{(2)} \overline{w}_{j} } = \overline{q} \\ \end{aligned} $$

and the boundary conditions are

37 $$ \begin{aligned} & \left[ { - \sum\limits_{j = 1}^{N} {A_{ij}^{(3)} \overline{w}_{j} } - K_{3} \sum\limits_{j = 1}^{N} {A_{ij}^{(1)} \overline{w}_{j} } \sum\limits_{j = 1}^{N} {A_{ij}^{(1)} \overline{w}_{j} } \sum\limits_{j = 1}^{N} {A_{ij}^{(1)} \overline{w}_{j} } } \right. \\ & \quad \left. { + \overline{V}_{p} \sum\limits_{j = 1}^{N} {A_{ij}^{(1)} \overline{w}_{j} } - K_{2} \sum\limits_{j = 1}^{N} {A_{ij}^{(1)} \overline{w}_{j} } \sum\limits_{j = 1}^{N} {A_{ij}^{(2)} \overline{w}_{j} } } \right]\delta \overline{w} |_{0}^{1} = 0 \\ \end{aligned} $$

38 $$ \left[ {\sum\limits_{j = 1}^{N} {A_{ij}^{(2)} \overline{w}_{j} } + K_{4} \sum\limits_{j = 1}^{N} {A_{ij}^{(1)} \overline{w}_{j} } \sum\limits_{j = 1}^{N} {A_{ij}^{(1)} \overline{w}_{j} } + K_{5} \overline{V}_{p} - \overline{V}_{f} } \right]\delta \frac{{{\text{ d}}\overline{w} }}{{{\text{ d}}\xi }}|_{0}^{1} = 0 $$

The discrete control equations and boundary conditions are solved iteratively.

## The electromechanical stimulation behaviors of piezoelectric beam

In this study, P(VDF-TrFE) is selected as the response layer and the driving layer since it has high dielectric constant, good piezoelectric properties, corrosion resistance. PDMS is selected as the intermediate elastic layer due to its characteristics of stable mechanical properties, biocompatibility and high transparency. For P(VDF-TrFE), $c_{1111} = 3.7{\text{ GPa}}$,$a_{33} = 1.38 \times 10^{10} {\text{ Nm}}^{2} /C^{2}$,$\varepsilon_{0} = 8.854 \times 10^{ - 12} {\text{ F}} /m$, $f_{3311} = - 179{\text{ Nm}} /C$,$d_{311} = 1.0212 \times 10^{9} {\text{ N}} /C$ and its material length scale parameter is assumed to be 1 μm. For PDMS, $c_{1111}^{e} = 2{\text{ MPa}}$ and its material length scale parameter is also assumed to be 1 μm. Therefore, the nonlinear bending of the piezoelectric micro-beam subjected to a drive voltage *V* = 10 V are solved by applying DQM in which the normalized Gauss–Chebyshev-Lobatto points $\varepsilon (i) = \frac{1}{2}[1 - \cos (\frac{i - 1}{{N - 1}}\pi )](i = 1,2,...,N)$ are used to generate the DQM point system with setting *N* = 30. In the numerical solution, after changing the number of discrete points and iterations, the error is very small and *N* = 30 satisfies accuracy.

Based on the extended dielectric theory, a static bending beam model is carried out using variational method, and the governing equation and all possible boundary conditions are determined. The static analysis of the micro-beam is carried out, and the relationship between the deflection and the electric potential is determined. The model can be directly applied to regulate the electromechanical stimulation of cell bionic culture. The results of the present model will reduce to that of pure flexoelectric effect when the piezoelectric effect is ignored. And the result of this model will degenerate to that of pure piezoelectric effect when the flexoelectric effect is ignored.

For cantilevered beam, the boundary conditions are

39 $$ \overline{w}_{1} = 0 $$

40 $$ \sum\limits_{j = 1}^{N} {A_{1j}^{(1)} \overline{w}_{j} } = 0 $$

41 $$ \begin{aligned} & - \sum\limits_{j = 1}^{N} {A_{Nj}^{(3)} \overline{w}_{j} } - K_{3} \sum\limits_{j = 1}^{N} {A_{Nj}^{(1)} \overline{w}_{j} } \sum\limits_{j = 1}^{N} {A_{Nj}^{(1)} \overline{w}_{j} } \sum\limits_{j = 1}^{N} {A_{Nj}^{(1)} \overline{w}_{j} } \\ & + \;\overline{V}_{p} \sum\limits_{j = 1}^{N} {A_{Nj}^{(1)} \overline{w}_{j} } - K_{2} \sum\limits_{j = 1}^{N} {A_{Nj}^{(1)} \overline{w}_{j} } \sum\limits_{j = 1}^{N} {A_{Nj}^{(2)} \overline{w}_{j} } = 0 \\ \end{aligned} $$

42 $$ \sum\limits_{j = 1}^{N} {A_{Nj}^{(2)} \overline{w}_{j} } + K_{4} \sum\limits_{j = 1}^{N} {A_{Nj}^{(1)} \overline{w}_{j} } \sum\limits_{j = 1}^{N} {A_{Nj}^{(1)} \overline{w}_{j} } + K_{5} \overline{V}_{p} - \overline{V}_{f} = 0 $$

For simply supported beam, the boundary conditions are

43 $$ \overline{w}_{1} = 0 $$

44 $$ \sum\limits_{j = 1}^{N} {A_{1j}^{(2)} \overline{w}_{j} } + K_{4} \sum\limits_{j = 1}^{N} {A_{1j}^{(1)} \overline{w}_{j} } \sum\limits_{j = 1}^{N} {A_{1j}^{(1)} \overline{w}_{j} } + K_{5} \overline{V}_{p} - \overline{V}_{f} = 0 $$

45 $$ \sum\limits_{j = 1}^{N} {A_{Nj}^{(2)} \overline{w}_{j} } + K_{4} \sum\limits_{j = 1}^{N} {A_{Nj}^{(1)} \overline{w}_{j} } \sum\limits_{j = 1}^{N} {A_{Nj}^{(1)} \overline{w}_{j} } + K_{5} \overline{V}_{p} - \overline{V}_{f} = 0 $$

46 $$ \overline{w}_{N} = 0 $$

**Figure 2:**
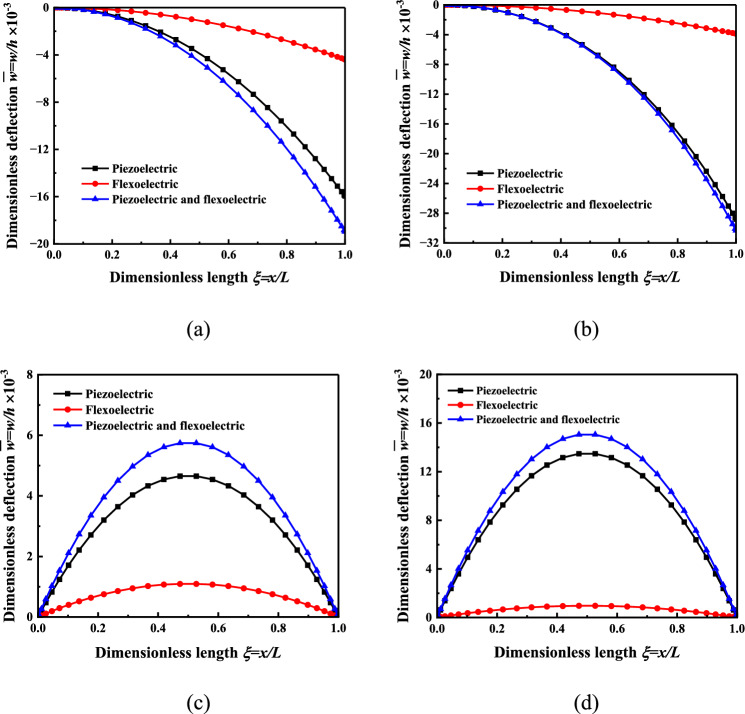
Nonlinear bending deflection distribution of the three-layer micro-beam. (**a**) Cantilever beam *H* = 2 μm. (**b**) Cantilever beam* H* = 5 μm*,* (**c**) Simply supported beam *H* = 2 μm*,* (**d**) Simply supported beam *H* = 5 μm.

**Figure 3:**
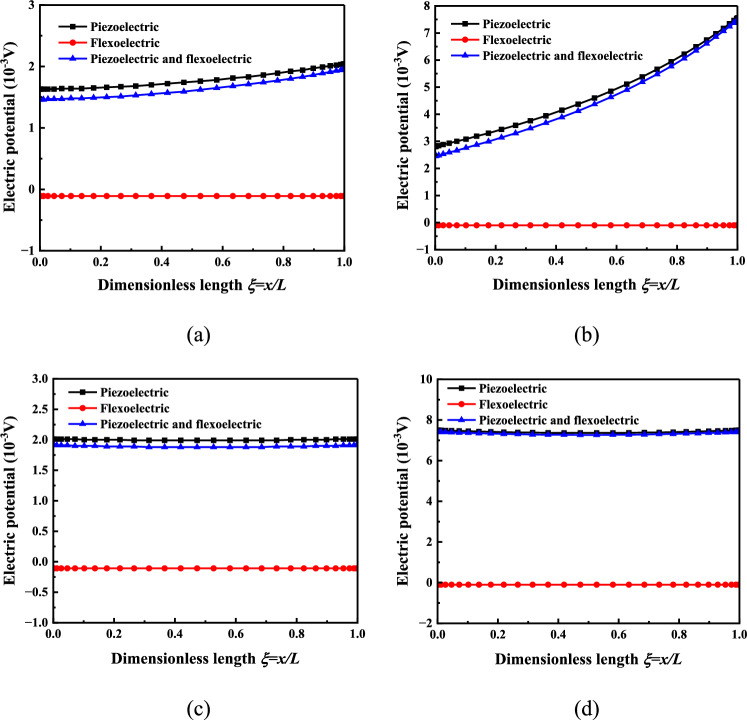
Surface electric potential distribution of the nonlinear bending response layer of the three-layer micro-beam (**a**) Cantilever beam *H* = 2 μm, (**b**) Cantilever beam* H* = 5 μm, (**c**) Simply supported beam *H* = 2 μm, (**d**) Simply supported beam *H* = 5 μm.

In order to investigate the mechanical force and electrical stimulations applied to the cells, numerical results are carried out based on the electromechanical stimulation regulating model. The geometric shape of the laminated micro-beam is *b* = *h* = 1 μm*, L* = 40*H* with *H* = *h* + 2*t* denoting the total thickness of laminated beam. Under the action of a pure voltage load *V* = 10 V, the dimensionless bending deflection of the cantilever micro-beam and the simply supported micro-beam is shown in Fig. 2. The deflection of the micro-beam is analyzed in three aspects: only considering piezoelectricity, only considering flexoelectricity, and considering both piezoelectricity and flexoelectricity. The electric potential distribution of the micro-beam response layer under the same conditions is shown as Fig. 3.

It can be seen from Fig. 2 that the dimensionless bending deflection of the micro-beam considering both the piezoelectric effect and the flexoelectric effect increases with the increase of the beam thickness, but in comparison to the dimensionless bending deflection of the micro-beam considering only the piezoelectric effect, the gap decreases as the thickness of the beam increases. That is, the flexoelectric effect is size-dependent. The larger the beam thickness, the smaller the contribution of the flexoelectric effect, and even this can be ignored. In the smaller size, the influence of the flexoelectric effect is significant, which is a non-negligible factor in the study of electromechanical coupling characteristic. When only the piezoelectric effect is considered, the deflection increases with the increase of the beam thickness, which is consistent with the results of piezoelectric elastic beam studied by Przybylski^13^. When only the flexoelectric effect is considered, the deflection decreases with the increase of the beam thickness, which is consistent with the results of the bending piezoelectric beam studied by Yan^32^.

Figure 3 shows the electric potential distribution of the micro-beam response layer, which is the main reference for the electrical stimulation microenvironment of cell bionic culture. The electric potential of the micro-beam response layer is negative when only the flexoelectric effect is considered, and the electric potential of the micro-beam response layer is positive when only the piezoelectric effect is considered, so that the electric potential of the micro-beam response layer considering both the piezoelectric effect and the flexoelectric effect is smaller than that of the micro-beam response layer considering only the piezoelectric effect. The electric potential of the micro-beam response layer when only the piezoelectric effect is considered is much larger than that when only the flexoelectric effect is considered, and as the thickness of the micro-beam increases, the electric potential of the micro-beam response layer also increases. This provides an important basis for constructing the electrical stimulation microenvironment in cell bionic culture.

**Figure 4:**
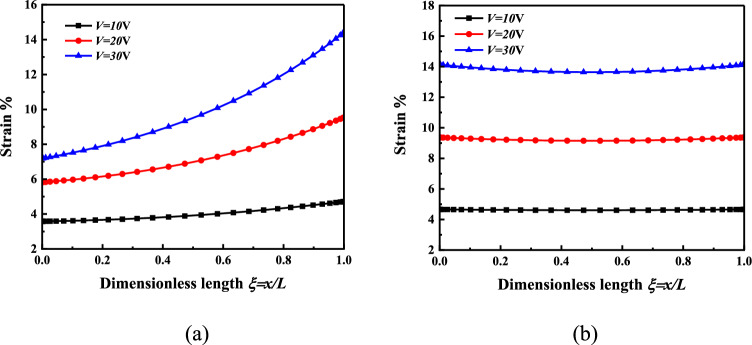
The strain distribution of the nonlinear bending response layer of the microbeam is *H* = 2 μm*, V* = 10 V *or V* = 20 V *or V* = 30 V (**a**) Cantilever beam (**b**) Simply supported beam.

When *H* = *2*μm, *V* = 10V *or V* = 20V *or V* = 30V, the surface strain of the micro-beam response layer is analyzed, as shown in Fig. 4.

Figure 4 shows the strain distribution on the surface of the micro-beam response layer to provide mechanical stimulation to the cells. It can be found that at the same thickness, the strain of the cantilever beam and the simply supported beam response layer increases with the increase of voltage. It can be found from Fig. 4a that the strain in the length direction of the cantilever beam is increasing at the same voltage. According to the change in deflection of the cantilever beam under the same voltage in Fig. 2, it is not difficult to find that the deflection changes more and more along the length direction of the cantilever beam. Therefore, along the length direction of the cantilever beam, the surface strain of the cantilever beam response layer also increases. It can be obtained from Fig. 4b that under the same voltage, the strain at the two fixed ends of the simply supported beam is larger than that in the middle. According to the change of deflection of a simply supported beam under the same voltage in Fig. 2, it can be obtained that along the length direction of the simply supported beam, the change of deflection decreases first and then increases. Therefore, along the length direction of a simply supported beam, the surface strain of the response layer also decreases first and then increases. The strain provides mechanical stimulation, and the cells attach to the response layer. The strain in the response layer makes the cells produce a corresponding strain. This is of great significance for constructing the mechanical microenvironment of cell bionic culture.

**Figure 5:**
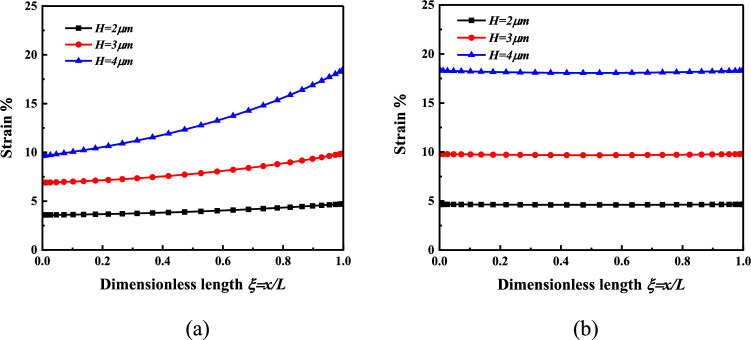
The strain distribution of the nonlinear bending response layer of the microbeam is *V* = 10 V*, H* = 2 μm *or H* = 3 μm or *H* = 4 μm (**a**) Cantilever beam (**b**) Simply supported beam.

When *V* = 10 V, *H* = *2* μm *or H* = *3* μm *or H* = *4* μm, the surface strain of the micro-beam response layer is analyzed, as shown in Fig. 5.

Figure 5 shows the strain distribution on the surface of the micro-beam response layer under different thicknesses. It can be found that under the same voltage, the strain on the surface of the cantilever beam and the simply supported beam response layer increases with the increase of the beam thickness. This provides a strong support for the selection of material thickness in the construction in cell bionic culture microenvironment.

**Figure 6:**
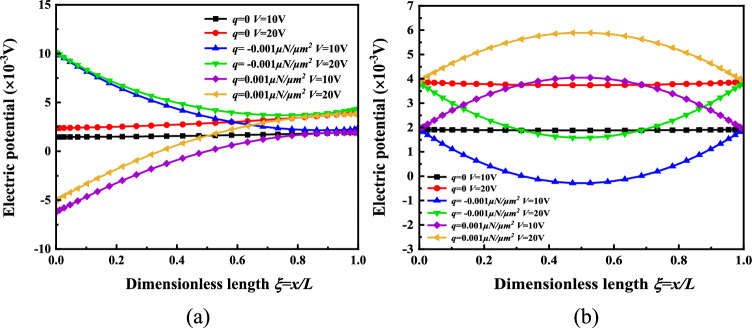
The electric potential distribution on the surface of the micro-beam response layer is *q* = *0, V* = 10 V *or V* = 20 V; *q* = -0.001 μN/μm^2^*, V* = 10 V or *V* = 20 V; *q* = 0.001 μN/μm^2^, *V* = 10 V *or V* = 20 V (**a**) Cantilever beam (**b**) Simply supported beam.

In order to further study the effects of the applied electrical load and mechanical load on the electric potential change of the micro-beam response layer, the following graphic analysis was carried out. When *H* = *2 *μm, the electric potential of the micro-beam response layer is *q* = *0, V* = *10* V *or V* = 20 V; *q* = − 0.001 μN/μm^2^*, V* = *10* V *or V* = 20 V; *q* = 0.001 μN/μm^2^*, V* = *10* V *or V* = 20 V, as shown in Fig. 6. It can be found from Fig. 6 that when the same load is applied, the electric potential on the surface of the cantilever beam and the simply supported beam response layer increases with the increase in the applied voltage. When the voltage is 10 V, the electric potential after applying the load *q = − *0.001 μN/μm^2^ and *q* = 0.001 μN/μm^2^ is relatively symmetrical to the electric potential without applying the load, and the same is true when the voltage is 20 V. The variation of the electric potential on the surface of the response layer with the applied force and the electrical load provides a great help for the controllability of the electrical stimulation microenvironment required for cell bionic culture.

**Figure 7:**
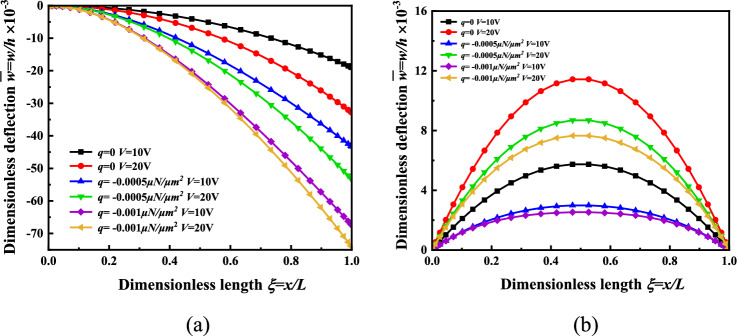
The nonlinear bending deflection distribution of the micro-beam is *q* = *0, V* = *10* V *or V* = 20 V; *q* = -0.0005 μN/μm^2^, *V* = 10 V or *V* = 20 V; *q* = -0.001 μN/μm^2^, *V* = 10 V or *V* = 20 V. (**a**) Cantilever beam, (**b**) Simply supported beam.

In order to further study the effects of the applied electrical load and mechanical load on the deflection of the micro-beam, the following graphic analysis was carried out. When *H* = *2*μm, the deflection of the micro-beam is *q* = *0, V* = *10* V *or V* = 20 V; *q* = − 0.0005 μN/μm^2^*, V* = *10* V *or V* = 20 V; *q* = − 0.001 μN/μm^2^*, V* = *10* V *or V* = 20 V, as shown in Fig. 7.

Figure 7a shows that when the applied load is constant, the deflection of the cantilever beam increases with increasing applied voltage. When the applied voltage is constant, the deflection of the cantilever beam increases with the increase of the applied load. And when the applied load is greater, the deflection of the cantilever beam decreases with the increase of voltage. Figure 7b shows that at constant applied load, the deflection of the simply supported beam increases with increasing applied voltage; at constant applied voltage, the deflection of the simply supported beam decreases with increasing applied load. And when the applied load is larger, the deflection of the simply supported beam decreases as the voltage increases. Regarding the sensitivity analysis, we consider the sensitivity of the electrical load and the force load. In the case of the same other parameters, when the force load is from − 0.0005 to − 0.001 μN/μm^*2*^, the ratio of the change of the deflection of the micro beam to the change of the force load is 48,000; similarly, when the electric load is from 10 to 20V, the ratio of the deflection change of the micro-beam to the change of the electric load is 0.5. Therefore, it is suitable for coarse-tuning when applying a force load and for fine-tuning when applying an electrical load. The deflection of the micro-beam provides a great help for the regulation of the mechanical stimulation microenvironment required for cell bionic culture according to the change law of the applied mechanical and electrical load.

## Conclusions

In this paper, a laminated piezoelectric micro-beam including driving layer, elastic layer and response layer is used to construct the electromechanical stimulation microenvironment of cell bionic culture. When an electric load is applied to the driving layer, the driving layer will drive the overall bending deformation of the laminated micro-beam under the piezoelectric effect. And, at the same time, the response layer induces the electric potential through the inverse piezoelectric effect, and the cells cultured on the surface of the response layer are stimulated by mechanical force and electrical stimulation. In order to regulate the electromechanical stimulation microenvironment of cell bionic culture, a size-dependent model of the three-layer piezoelectric micro-beam is established. The mechanical governing equations and boundary conditions are derived by using the variational principle, and then discretized through the differential quadrature method. To analyze the size-dependent bending behavior of the laminated micro-beam, two boundary value problems involving a cantilever beam and a simply supported beam are solved independently. The combination of the differential quadrature method with the iterative approach proves effective for this analysis.

The numerical results analyzed the microscale effect and the mechanical force and electrical stimulations applied to the cells in detail. For the cantilever beam and the simply supported beam, the same flexoelectric responses exist. The flexoelectric effect is related to the dimensionless thickness of the micro-beam, and as the size decreases, the flexoelectric effect becomes more obvious. The strain and electrical potential at the surface of the response layer increase with the increase of applied electrical load. The laminated micro-beam model established in this study can be applied to the biomedical field to improve the quality of cell culture by regulating force and electrical stimulation. The results can be helpful to provide accurate mechanical and electrical stimulation in cell bionic culture by applying accurate driving voltage and force.

## Electronic supplementary material

Below is the link to the electronic supplementary material.

Supplementary Information.

## Data Availability

Data is available on request from the corresponding author.
