# Supplementary material for: An electromechanical stimulation regulating model with flexoelectric effect of piezoelectric laminated micro-beam for cell bionic culture
Source: Sci Rep. 2024 Mar 13;14:6130. doi: 10.1038/s41598-024-56708-9 (PMC11636837; doi:10.1038/s41598-024-56708-9)
Supplement: Supplementary file 1 — Supplementary Information. [file 41598_2024_56708_MOESM1_ESM.docx]

**Appendix A**

The parameters *a*_1_ ~ *a*_6_ in Eqs. - are shown as

$a_{1}=2c_{1111}(\frac{h^{2}t}{4}+\frac{ht^{2}}{2}+\frac{t^{3}}{3})+2g_{311311}t+\frac{c_{1111}^{e}h^{3}}{12}+g_{311311}^{e}h$ (A.1)

$a_{2}=\frac{d_{311}^{2}t}{2a_{33}}+\frac{d_{311}^{2}t}{2b_{3333}\lambda^{2}}-3(c_{1111}t+\frac{c_{1111}^{e}h}{2})$ (A.2)

$\begin{matrix} a_{3}=\frac{d_{311}^{2}t(\text{h}+t)-2d_{311}f_{3311}t(1+2b_{3333}\varepsilon_{0}\lambda^{2})}{2a_{33}b_{3333}\varepsilon_{0}\lambda^{2}} \\ +(\frac{2d_{311}^{2}}{b_{3333}\lambda^{4}}-\frac{2d_{311}f_{3311}}{b_{3333}\lambda^{3}}+\frac{d_{311}f_{3311}h}{b_{3333}\lambda^{2}}+\frac{f_{3311}^{2}(h-2t)}{b_{3333}\lambda})\frac{2-e^{\lambda t}-e^{-\lambda t}}{e^{-\lambda t}-e^{\lambda t}} \end{matrix}$ (A.3)

$a_{4}=\frac{d_{311}^{2}t}{2a_{33}}+\frac{d_{311}^{2}t}{2b_{3333}\lambda^{2}}-(c_{1111}t+\frac{c_{1111}^{e}h}{2})$ (A.4)$\begin{matrix} a_{5}=-\frac{2f_{3311}^{2}t(1+2b_{3333}\varepsilon_{0}\lambda^{2})-f_{3311}d_{311}t(h+t)}{2a_{33}b_{3333}\varepsilon_{0}\lambda^{2}} \\ +\frac{2d_{311}f_{3311}t(1+2b_{3333}\varepsilon_{0}\lambda^{2})-d_{311}^{2}t(h+t)}{4a_{33}b_{3333}\varepsilon_{0}\lambda^{2}}(h+t) \\ -\frac{d_{311}f_{3311}t}{b_{3333}\lambda^{2}}(h+t)-\frac{2f_{3311}^{2}t}{b_{3333}\lambda^{2}}+(\frac{2d_{311}f_{3311}}{b_{3333}\lambda^{4}}+\frac{f_{3311}^{2}h}{b_{3333}\lambda^{2}})\frac{2-e^{\lambda t}-e^{-\lambda t}}{e^{-\lambda t}-e^{\lambda t}} \\ +(2c_{1111}t-\frac{2d_{311}^{2}t}{b_{3333}\lambda^{2}})(\frac{h^{2}}{4}+\frac{ht}{2}+\frac{t^{2}}{3})+2g_{311311}t+\frac{c_{1111}^{e}h^{3}}{12}+g_{311311}^{e}h \\ -\frac{2d_{311}f_{3311}t}{b_{3333}\lambda^{3}}\frac{2+\lambda^{2}[(-h/(2\lambda)-t/\lambda-1/\lambda^{2})e^{-\lambda t}+(h/(2\lambda)+t/\lambda-1/\lambda^{2})e^{\lambda t}]}{e^{-\lambda t}-e^{\lambda t}} \\ +(\frac{f_{3311}^{2}h}{b_{3333}}-\frac{2d_{311}^{2}}{b_{3333}\lambda^{3}})\frac{[-h/(2\lambda)+(h/(2\lambda)+t/\lambda-1/\lambda^{2})e^{\lambda t}+1/\lambda^{2}](1-e^{-\lambda t})}{e^{-\lambda t}-e^{\lambda t}} \\ -(\frac{2f_{3311}d_{311}}{b_{3333}\lambda^{2}}+\frac{f_{3311}d_{311}h}{b_{3333}\lambda})\frac{[-h/(2\lambda)+(h/(2\lambda)+t/\lambda+1/\lambda^{2})e^{-\lambda t}-1/\lambda^{2}](1-e^{\lambda t})}{e^{-\lambda t}-e^{\lambda t}} \end{matrix}$(A.5)

$\begin{matrix} a_{6}=\frac{f_{3311}d_{311}t}{2a_{33}}-\frac{d_{311}^{2}t}{4a_{33}}(h+t)+\frac{f_{3311}d_{311}t}{2b_{3333}\lambda^{2}}+\frac{d_{311}^{2}t}{4b_{3333}\lambda^{2}}(h+t) \\ -\frac{f_{3311}^{2}}{b_{3333}}\frac{[-h/(2\lambda)+(h/(2\lambda)+t/\lambda+1/\lambda^{2})e^{-\lambda t}-1/\lambda^{2}](1-e^{\lambda t})}{e^{-\lambda t}-e^{\lambda t}} \\ -\frac{f_{3311}^{2}}{b_{3333}}\frac{[-h/(2\lambda)+(h/(2\lambda)+t/\lambda-1/\lambda^{2})e^{\lambda t}+1/\lambda^{2}](1-e^{-\lambda t})}{e^{-\lambda t}-e^{\lambda t}} \end{matrix}$ (A.6)
